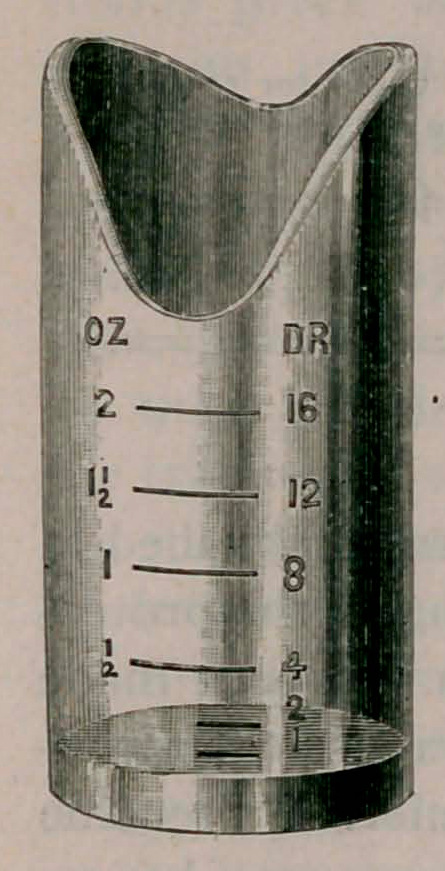# The McKesson & Robbins Nose Cup

**Published:** 1902-04

**Authors:** William James Evans

**Affiliations:** New York


### NEW INSTRUMENT.

### The McKesson & Robbins Nose Cup.

A NEW ADDITION TO THE TOILET.—NO MORE MOUTH BREATHING.

By WILLIAM JAMES EVANS, New York.

AN interesting paper might be written on the changes that would take place if the world were suddenly deprived of all its tooth brushes and other toilet articles.

The clinical evidence resulting from neglect of the teeth formerly kept in a healthy condition, would however be no more alarming than the conditions which have obtained prior to the recent introduction of the McKesson & Robbins nose cup.

A very small percentage of men, and not more than 5 per cent. of women, breathe properly. About 24 per cent. of the people die of throat and lung diseases. It is not disputed that over half of this number would live to be much older and probably die of other diseases if they took better care of their nostrils. Mouth breathers are invariably people with poor digestion, who show all the evidences of malnutrition as a consequence of the malocclusion of the teeth, which has its primary cause in obstruction of the nasal passages.

We have always known that only the uncleanly breathe with open mouths, but we have not formerly had a device so simple and effective with which to form one of the most necessary habits in connection with the toilet. The constant irritation of the mucous membrane of the nostrils, by the infected dust of large cities and unclean buildings, produces the local heat, which makes the benign and otherwise healing mucus dry in patches in the nostrils and at the back of the mouth breather's throat to such an extent that naso-pharyngeal lavage is imperative.

Irrigation of the nasal passages with the nose cup which I have devised, and which is herewith illustrated, will soon change the pathological to normal structures, and transform the mouth breather to the nose breather. This method should become as much a part of the toilet as the use of the tooth brush.

Fortunately this irrigation prompts the flow of healthy mucus which has bactericidal power and, in covering the denuded portions of the mucous membrane, gives the best protection and the most comfortable relief. The nostrils should not be violently blown at any time and not at all when there is either water or saline solution in them, but can more safely be cleared after waiting for the flow of normal secretion, which after all is nature's remedy when conditions are favorable.

The illustration gives an understanding of the shape and size of the cup. It is 3 inches high by 1¾ in diameter, made of strong glass and can be carried in the pocket or traveling bag.

The price is but 25 cents and it is supplied by all druggists.

## Figures and Tables

**Figure f1:**